# Determining the optimal screening interval for type 2 diabetes mellitus using a risk prediction model

**DOI:** 10.1371/journal.pone.0187695

**Published:** 2017-11-14

**Authors:** Andrei Brateanu, Thomas Barwacz, Lei Kou, Sihe Wang, Anita D. Misra-Hebert, Bo Hu, Abhishek Deshpande, Nana Kobaivanova, Michael B. Rothberg

**Affiliations:** 1 Medicine Institute, Cleveland Clinic, Cleveland OH, United States of America; 2 Department of Medicine, University Hospitals, Cleveland OH, United States of America; 3 Quantitative Health Sciences, Cleveland Clinic, Cleveland OH, United States of America; 4 Pathology and Laboratory Medicine Institute, Cleveland Clinic, Cleveland OH, United States of America; Baylor College of Medicine, UNITED STATES

## Abstract

**Background:**

Progression to diabetes mellitus (DM) is variable and the screening time interval not well defined. The American Diabetes Association and US Preventive Services Task Force suggest screening every 3 years, but evidence is limited.

The objective of the study was to develop a model to predict the probability of developing DM and suggest a risk-based screening interval.

**Methods:**

We included non-diabetic adult patients screened for DM in the Cleveland Clinic Health System if they had at least two measurements of glycated hemoglobin (HbA1c), an initial one less than 6.5% (48 mmol/mol) in 2008, and another between January, 2009 and December, 2013. Cox proportional hazards models were created.

The primary outcome was DM defined as HbA1C greater than 6.4% (46 mmol/mol). The optimal rescreening interval was chosen based on the predicted probability of developing DM.

**Results:**

Of 5084 participants, 100 (4.4%) of the 2281 patients with normal HbA1c and 772 (27.5%) of the 2803 patients with prediabetes developed DM within 5 years. Factors associated with developing DM included HbA1c (HR per 0.1 units increase 1.20; 95%CI, 1.13–1.27), family history (HR 1.31; 95%CI, 1.13–1.51), smoking (HR 1.18; 95%CI, 1.03–1.35), triglycerides (HR 1.01; 95%CI, 1.00–1.03), alanine aminotransferase (HR 1.07; 95%CI, 1.03–1.11), body mass index (HR 1.06; 95%CI, 1.01–1.11), age (HR 0.95; 95%CI, 0.91–0.99) and high-density lipoproteins (HR 0.93; 95% CI, 0.90–0.95). Five percent of patients in the highest risk tertile developed DM within 8 months, while it took 35 months for 5% of the middle tertile to develop DM. Only 2.4% percent of the patients in the lowest tertile developed DM within 5 years.

**Conclusion:**

A risk prediction model employing commonly available data can be used to guide screening intervals. Based on equal intervals for equal risk, patients in the highest risk category could be rescreened after 8 months, while those in the intermediate and lowest risk categories could be rescreened after 3 and 5 years respectively.

## Introduction

Diabetes Mellitus (DM) is a chronic disease associated with micro and macro vascular complications and multiple organ damage.[1] It is estimated that approximately 29 million Americans have DM, and 8.1 million of these are undiagnosed. In addition, 86 million Americans aged 20 years or older have prediabetes[2] and 70% of them will ultimately develop DM.[3] However, this progression can be stopped with medications and more importantly, with diet and lifestyle changes.[4]

Identifying asymptomatic diabetics and individuals at risk for developing DM may reduce the burden associated with this disease. While the United States Preventive Services Task Force (USPSTF) recommends glucose screening in overweight or obese adults aged 40 to 70 years,[5] the American Diabetes Association suggests that screening should be done in all adults beginning at age 45 years regardless of weight, or at any age when obesity or at least one DM risk factor is present.[6] Glucose abnormalities can be identified measuring the fasting plasma glucose, glycated hemoglobin (HbA1c), or with the oral glucose tolerance test. HbA1c has several advantages when compared to the other two tests: it measures chronic glycemic levels, does not require fasting or two hour testing, and correlates well with the risk of subsequent diabetic complications. In addition, it can be used to predict the subsequent DM risk.[7]

Previous models to predict the risk of developing DM in the general population have been designed in European patient populations,[8–12] or did not use HbA1c.[13, 14]

In addition, the optimal time for rescreening in patients without DM is not known. A few studies using mathematical modeling[15–18] or cohorts of Asian populations[19] suggested a time interval of 3 years for rescreening patients with normal glucose.

The objective of this study was to create a model to predict the risk of progression to DM in patients with normal glucose or pre-diabetes, based upon commonly available risk factors and determine the optimal time for rescreening an individual based on his or her risk.

## Research design and methods

### Design and setting

We conducted a retrospective cohort study in the Cleveland Clinic Health System (CCHS), a not-for-profit health system located in Cleveland, Ohio that includes an academic medical center, 8 community hospitals and 36 community-based practices. CCHS uses a common electronic health record system (Epic Systems Corporation, Inc.) allowing for the medical data to be easily extracted. The Cleveland Clinic serves an estimated population of >1.5 million people, with the majority (75%) of patients coming from the seven counties adjacent to Cleveland, and the rest from other parts of Unites States or from abroad. To be included, patients had to be 18 years or older, with at least two measurements of HbA1c, an initial one recorded between January 1 and December 31, 2008, and a subsequent one between January 1, 2009 and December 31, 2013, in the inpatient or outpatient setting. Exclusion criteria included any of the following prior to 2009: any HbA1c of 6.5% (48 mmol/mmol) or higher, a diagnosis of type 1 or 2 diabetes or gestational diabetes based on ICD-9 codes, and patients taking any glucose lowering medications or steroids. The Cleveland Clinic Institutional Review Board approved the study protocol.

### Potential predictors

The following factors are known to be independently associated with the development of type 2 diabetes: blood glucose level,[20] age,[21] race,[22] family history,[23] increased body mass index (BMI),[23] high blood pressure,[23] elevated levels of triglycerides or liver enzymes,[23] proteinuria,[24] and smoking.[25, 26] In addition, an increased level of high-density lipoproteins (HDL) is known to be associated with a decreased risk of DM.[23] Studies regarding varying levels of alcohol consumption as a risk factor for developing DM have been inconsistent.[27]

Therefore, the following variables were collected for each patient at baseline: demographic data (age, sex and race), alcohol consumption and cigarette smoking status, body mass index (BMI), treatment with statins or antihypertensive medications, family history of DM in a first degree relative. Laboratory data included: HbA1c, lipid fractions (HDL, low-density lipoproteins (LDL), triglycerides (TG)), liver enzymes (alanine aminotransferase (ALT)), and proteinuria, determined within 3 months before or after the first HbA1c measurement.

### Outcomes

The primary outcome of the study was progression to DM in patients with prediabetes or no DM, within five years of the initial HbA1c measurement. DM was diagnosed with at least one reading of HgbA1c greater than or equal to 6.5% (48 mmol/mol).

### Statistical analysis

Cox proportional hazards models were built to predict the risk of developing DM within five years of the initial HBA1c measurement. Based on the concept of equal treatment for equal risk, we estimated time intervals for screening among tertiles of risk, so that each tertile would be screened when 5% of the cohort developed DM. We chose a 5% probability as the threshold, because this is approximately equal to the proportion of people found to have DM when patients are initially screened based on the current ADA recommendations.[6, 15]

Cumulative incidence was plotted to depict the probability of developing DM over time. Univariate analysis was employed to evaluate the association of all covariates with the development of DM. For all regression models, candidate variables for multivariable analysis were evaluated using a stepwise selection procedure, with a P value of 0.05 from the univariate analysis as the threshold for entry into the model. A multivariate Cox proportional hazard model was built, to predict the risk of developing DM within 5 years after the initial HBA1c measurement. Cubic spline models were built to relax model assumption. After the initial model was created, a stepdown method of backwards elimination was applied to create the reduced model, which had best prediction power. Then the model was internally validated using 1000 bootstrap iterations. Model discrimination was measured by the C index and calibration between predicted and observed probabilities was assessed visually via calibration plot.

## Results

A total of 5084 participants were included in the study, and 2803 (55.3%) had prediabetes at study enrollment. Average age was 58.3 ± 13.3 years, 60.3% were men, and the majority were Caucasians (83.3%). Approximately half of the patients were taking statins (46.3%) and/or antihypertensive medications (56%). Within 5 years of the initial HbA1c measurement, 872 (17.2%) patients developed DM, of which 100 (11.5%) had normal glucose and 772 (88.5%) had prediabetes at study enrolment (**Table 1**).

**Table 1:** Characteristics of the study population.

Variables	Total cohort	No DM within 5 years	Developed DM within 5 years	P value
	5084	4212	872	
	N (%) or Mean ± SD	N (%) or Mean ± SD	N (%) or Mean ± SD	
Age (years)				
	58.3 ± 13.3	58.1 ± 13.3	59.2 ± 13	0.02
Race*				
Caucasian	4185 (83.3)	3466 (83.2)	719 (83.5)	0.06
African American	499 (9.9)	402 (9.7)	97 (11.3)	
Other	342 (6.8)	297 (7.1)	45 (5.2)	
Sex				
Male	3064 (60.3)	2550 (60.5)	514 (59)	0.40
Active smoker^†^				
Yes	2147 (47.8)	1709 (46.2)	438 (55.1)	<0.001
Alcohol use^‡^				
Yes	2702 (63.8)	2282 (65.3)	420 (56.4)	<0.001
BMI^§^ (kg/m^2^)				
	30.2 ± 6.7	29.7 ± 6.5	32.5 ± 7.3	<0.001
Family history of DM^§§^^‖^				
Yes	1183 (23.6)	906 (21.7)	277 (32.9)	<0.001
Taking statins				
Yes	2356 (46.3)	1889 (44.8)	467 (53.6)	<0.001
Taking antihypertensive medications				
Yes	2845 (56)	2273 (54.0)	572 (65.6)	<0.001
Lipid profile (mg/dl)				
HDL^¶^	52.3 ± 15.6	53.4 ± 15.8	46.9 ± 13.6	<0.001
TG^#^	132.1 ± 85.3	126.5 ± 80.8	162.0 ± 100.6	<0.001
LDL**	109.8 ± 34.7	110.4 ± 34.2	106.5 ± 36.9	<0.01
Liver enzymes (IU/L)				
ALT^††^	27.1 ± 34.6	26.4 ± 36.1	30.8 ± 25.4	<0.001
HbA1c (%)				
Yes	5.7 ± 0.4	5.6 ± 0.4	6.0 ± 0.3	<0.001
HbA1c category				
Normal (<5.7)	2281 (44.9)	2181 (51.8)	100 (11.5)	<0.001
Prediabetes (5.7–6.4)	2803 (55.1)	2031 (48.2)	772 (88.5)	

Missing data for

*, 58 patients

^†^, 591 patients

^‡^, 847 patients

^§^, 213 patients

^§§^, 73 patients

^¶^, 1863 patients

^#^, 1872 patients

**, 1957 patients

^††^, 1466 patients

BMI, body mass index; DM, diabetes mellitus; HDL, high-density lipoproteins; TG, triglyceride; LDL, low-density lipoproteins; ALT, alanine aminotransferase; HbA1c, glycated hemoglobin.

Compared to patients with no DM within 5 years, patients who developed DM were older and had a higher BMI. Patients who developed DM were also more likely to be taking statins and antihypertensive medications, had higher levels of serum TG and lower of HDL.

**Fig 1:**
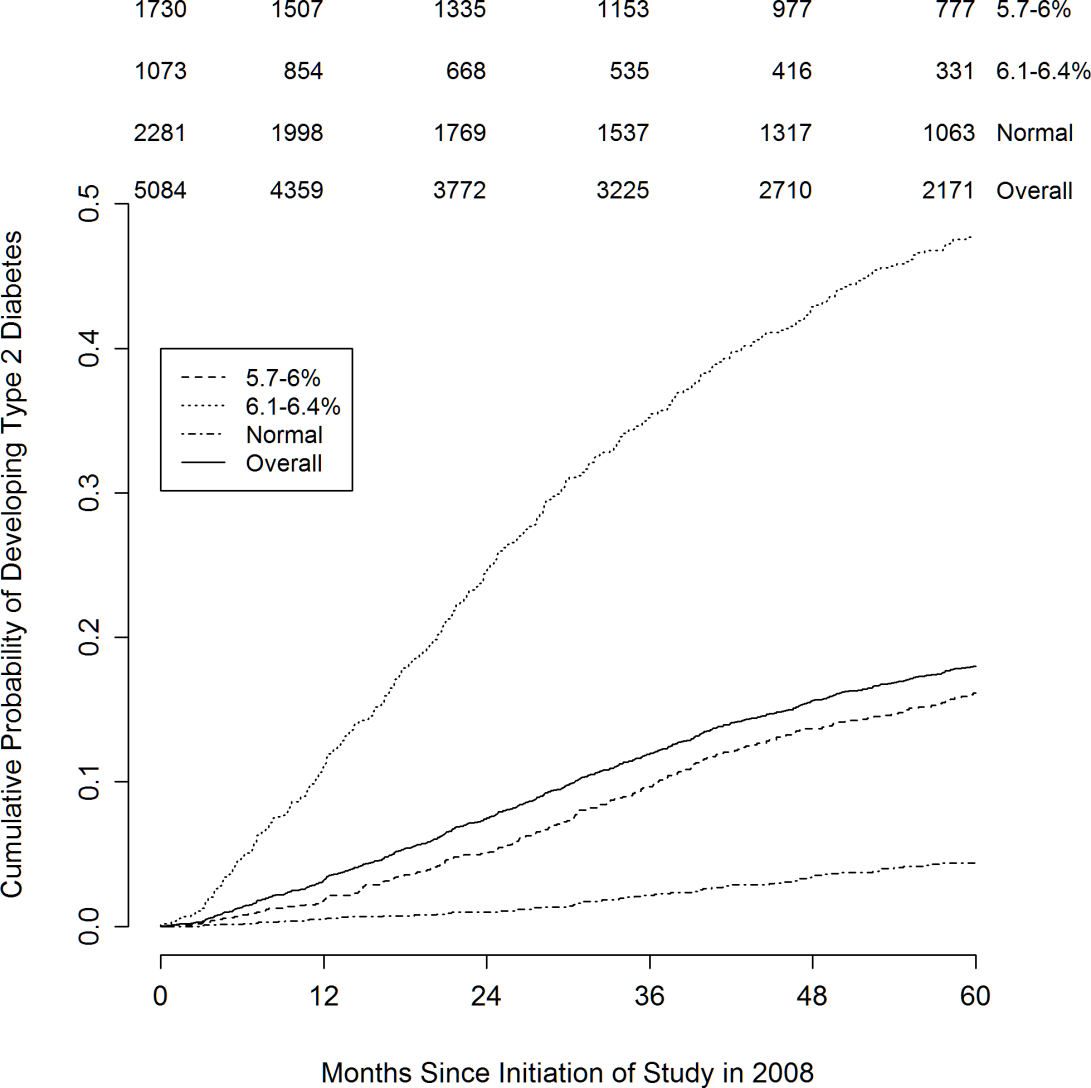
Cumulative probability of developing diabetes mellitus. Cumulative probability of 5% would be reached in 19 months for all patients, in 7 months for patients with HbA1c 6.1–6.4%, and in 26 months for patients with HbA1c 5.7–6.0%. Patients with HbA1c <5.7% won’t reach the 5% threshold within 5 years.

Based on HbA1c alone, patients had very different probabilities of developing DM at 3 years (**Fig 1**).

More than one third of patients with an initial HbA1c between 6.1% (43 mmol/mol) and 6.4% (46 mmol/mol) developed DM but only one in ten patients with HbA1c between 5.7% (39 mmol/mol) and 6.0% (42 mmol/mol), and fewer than 1 in 20 of those with normal glucose.

In the Cox proportional hazards model, the following factors were associated with the risk of developing DM within 5 years: age, BMI, active smoking, family history of DM, HDL, TG, ALT, and HbA1c. A family history of DM increased the risk by 30% (HR 1.31; 95%CI, 1.13–1.51), while a 0.1 unit increase in HbA1c increased the risk by 20% (HR per 0.1 units increase 1.20; 95%CI, 1.13–1.27) and smoking increased the risk by 18% (HR 1.18; 95%CI, 1.03–1.35). High-density lipoprotein was negatively associated with the risk of DM (HR 0.93; 95%CI, 0.90–0.95) (**Table 2**).

**Table 2:** Predictors of developing diabetes mellitus.

Variables	Hazard Ratio	Lower 95% CI	Upper 95% CI
Age (years)	0.95*	0.91	0.99
BMI (kg/m^2^)	1.06*	1.01	1.11
Active smoking: yes/no	1.18	1.03	1.35
Family history DM: yes/no	1.31	1.13	1.51
HDL (mg/dl)	0.93*	0.90	0.95
TG (mg/dl)	1.01*	1.00	1.03
ALT (IU/L)	1.07*	1.03	1.11
HbA1c (%)	1.20^†^	1.13	1.27

* Predicted change in the HR for a 5-unit increase in the predictor.

^†^, Predicted change in the HR for a 0.1-unit increase in the predictor.

BMI, body mass index; DM, diabetes mellitus; HDL, high-density lipoproteins; TG, triglyceride; ALT, alanine aminotransferase; HbA1c, glycated hemoglobin.

**Fig 2:**
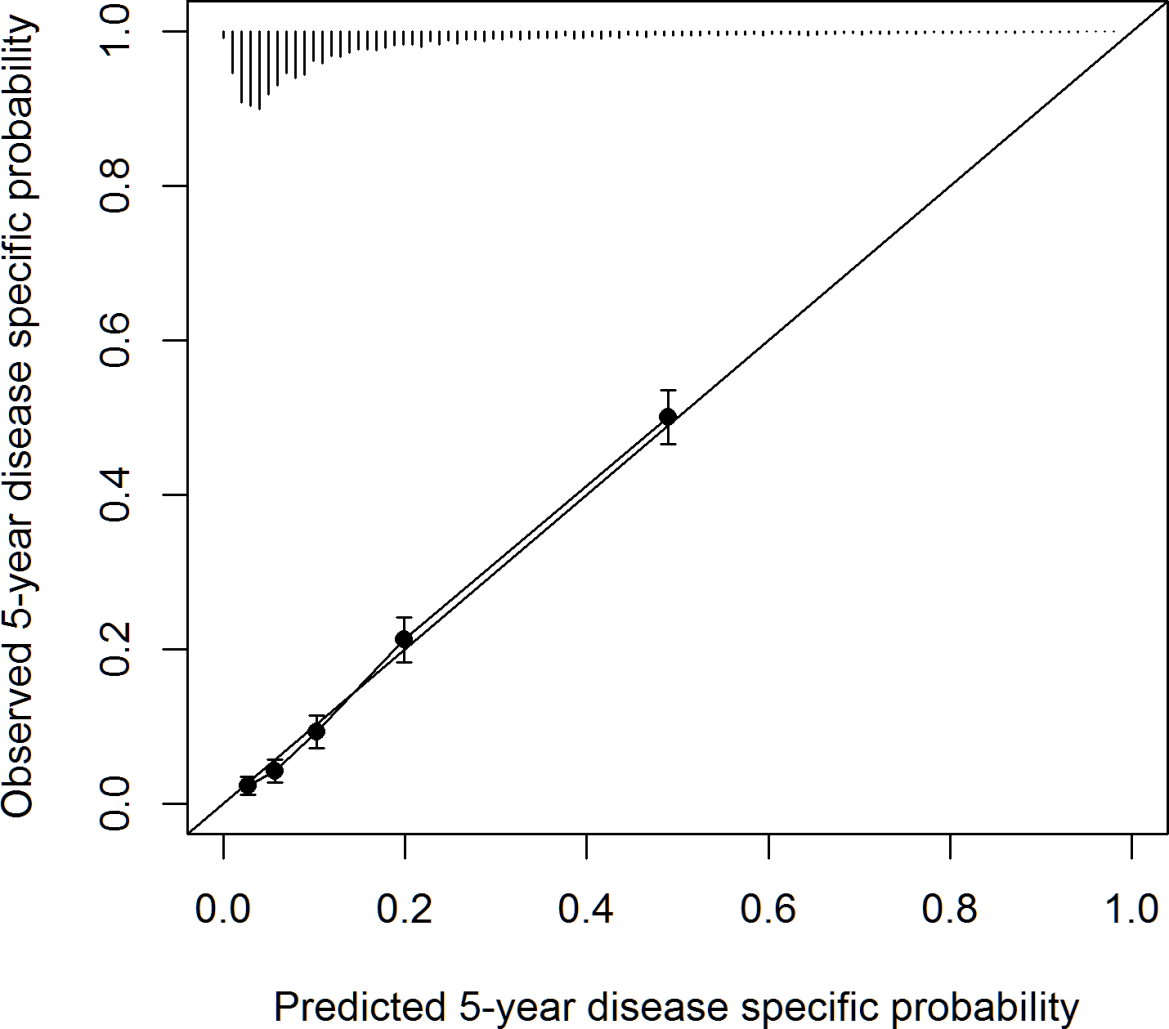
The calibration of the model, which measures the relationship between the model’s predicted probability against the actual probability. The final model, had a bootstrap bias-corrected c-statistic of 0.809 with a 95% CI (0.795, 0.823). Quintiles of 5-year risk ranged from 0.03–0.49 and were well calibrated.

The final model had a bootstrap bias-corrected c-statistic of 0.809 with a 95% CI (0.795, 0.823). **Fig 2** shows a calibration plot of observed vs. predicted risk of DM.

Quintiles of 5-year risk ranged from 0.03–0.49. The model showed good calibration with a ratio of observed to predicted risk of 1.02. An online calculator can be accessed at: http://riskcalc.org:3838/ab_diabetes_version2/

**Fig 3:**
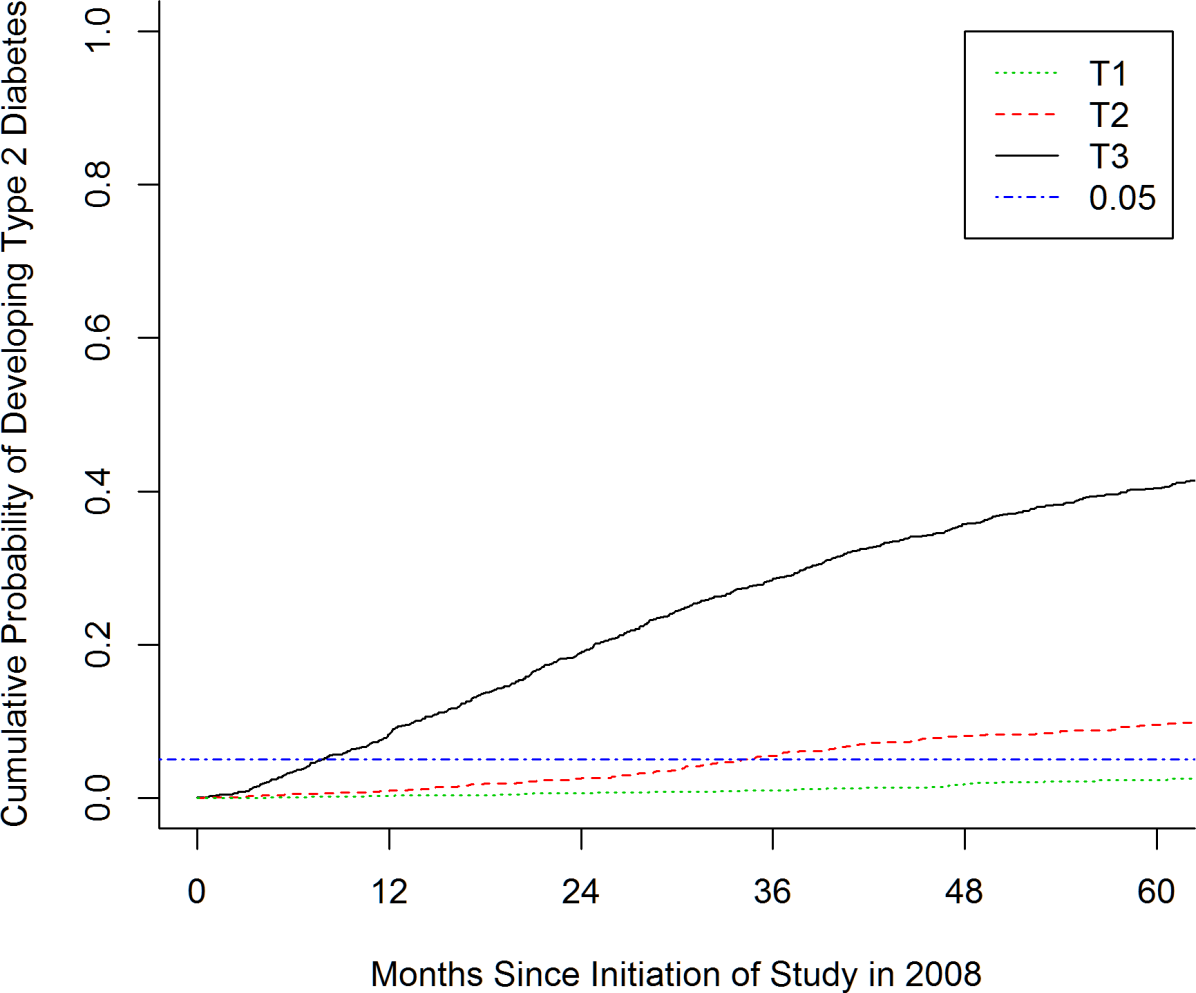
Cumulative probability of developing diabetes mellitus. Cumulative probability of 5% would be reached in 8 months for T3 and 35 months for T2. T1 won’t reach the 5% threshold.

Based on the model, we divided patients into tertiles of risk for DM. Five percent of patients in the highest risk tertile developed DM within 8 months, while it took 35 months for 5% of the middle tertile to develop DM. Only 2.4% percent of the patients in the lowest tertile developed DM within 5 years (**Fig 3**).

## Discussion and conclusions

In a large cohort of US adults with normal glucose or prediabetes we created a model to predict the time to develop DM within five years from the initial HgA1c testing, based on the risk of developing the disease. To our knowledge, this is a novel tool and has the advantage of using clinical predictors easily obtained when the initial HbA1C is measured. The model can stratify patients into risk categories to guide future testing intervals. More importantly, our model has been developed in already screened patients, and not in the general population, like previous models.

Assuming that patients should be screened when their risk is 5% or higher, patients in the highest risk category should be rescreened after only 8 months, while those in the lowest risk category could go for 5 years or more.

Screening the US population for DM, following the USPSTF[5] or ADA[6] guidelines will identify a significant number of prediabetics. Most patients with prediabetes will not progress quickly to DM, and progression can be slowed or even prevented with lifestyle or pharmacological interventions.[28] Risk stratification provides 2 potential benefits for these patients. First, it identifies those most likely to progress to DM, for whom intensive lifestyle therapy or treatment with metformin would be most indicated. Second, it allows for more efficient follow-up screening. Determining the appropriate screening interval requires weighing the benefits of early detection against the costs of frequent monitoring. The USPSTF currently recommends that individuals with an initial normal glucose should be re-screened every 3 years.[5] Applying this interval uniformly may result in over-screening of low-risk patients and under-screening of high-risk patients. Delaying the diagnosis and treatment of diabetic patients would be associated with an increase in cardiovascular events and all-cause mortality.[18] Alternatively, based on the concept of equal screening for equal risk, the screening interval for some individuals should be as short as 8 months while for others it might be longer than 5 years. Because of the harms associated with delayed diagnosis, it would probably not be prudent to wait more than 5 years, even though only a small percentage of low-risk patients would screen positive at that point.

Our study has a few limitations. It is based on a retrospective chart review, and the results may be subjected to bias and errors. We don’t know if the population who was screened at baseline is different from the general population and thus, validation in other medical settings would be needed. Patients taking steroids were excluded from the study due to the heterogeneity of doses, duration and individual indication for corticosteroid therapy. In addition, we were not able to identify and include other risk factors that might influence the risk of developing DM, such as waist circumference, physical activity, and diet.[8]

In conclusion, we created a tool to risk stratify non-diabetic patients that can be used in routine clinical practice. Because it is not practical for individual clinicians to seek out an external website, enter the patient’s data, and choose the screening interval, the tool may be best integrated into the electronic health record (EHR), where it could automatically generate a personalized screening interval based on risk. Physicians could then receive automatic reminders to monitor more frequently (e.g. every 8 months) patients with a higher risk of developing DM. Reminders would be sent less frequently (e.g. every 3, or 5 years) for patients with intermediate and low risk, respectively. In medical practices where the tool cannot be practically integrated in the EHR, the HbA1c alone can be used to determine the frequency of monitoring. Patients with HbA1c between 6.1% (43 mmol/mol) and 6.4% (46 mmol/mol) could be screened every 7 months, those with HbA1c between 5.7% (39 mmol/mol) and 6% (42 mmol/mol) every 2 years and those below 5.7% (39 mmol/mol) every 5 years.
